# Gut microbiota differences linked to weight gain and ART in people living with HIV are enterotype specific and minor compared to the large differences linked to sexual behavior

**DOI:** 10.3389/fcimb.2025.1568352

**Published:** 2025-05-08

**Authors:** Jan Kehrmann, Alireza Dostmohammadi, Anna-Lena Stumpf, Lara Best, Leah Consten, Hannah Sievert, Felix Maischack, Stefanie Sammet, Sarah Albayrak-Rena, Ann-Kathrin Doerr, Katharina Bohlen, Otgonzul von Velsen, Ivana Kraiselburd, Christina B. Karsten, Farnoush Farahpour, Folker Meyer, Stefan Esser, Jan Buer

**Affiliations:** ^1^ Institute of Medical Microbiology, University Hospital Essen, University of Duisburg-Essen, Essen, Germany; ^2^ Bioinformatics and Computational Biophysics, Faculty of Biology and Centre for Medical Biotechnology (ZMB), University of Duisburg-Essen, Essen, Germany; ^3^ Department of Dermatology, University Hospital Essen, University of Duisburg-Essen, Essen, Germany; ^4^ Institute for Artificial Intelligence in Medicine, University Hospital Essen, University of Duisburg-Essen, Essen, Germany; ^5^ Department of Gastroenterology, Hepatology and Transplantational Medicine, University Hospital Essen, Faculty of Medicine, University of Duisburg-Essen, Essen, Germany; ^6^ Institute of Medical Informatics, Biometrics, and Epidemiology, University Hospital Essen, University of Duisburg-Essen, Essen, Germany; ^7^ Center for Clinical Trials, University Hospital Essen, University of Duisburg-Essen, Essen, Germany; ^8^ Institute for the Research on HIV and AIDS-associated Diseases, University Hospital Essen, University of Duisburg-Essen, Essen, Germany; ^9^ Institute of Cell Biology (Tumor Research), University Hospital Essen, University of Duisburg-Essen, Essen, Germany

**Keywords:** HIV, gut microbiome, weight gain, art, dolutegravir, bictegravir, tenofovir

## Abstract

**Introduction:**

Specific antiretroviral therapy (ART) regimens are associated with weight gain in people living with HIV (PLWH). Gut microbiota is involved in weight gain in humans and animals. Human gut microbiota can be classified into enterotypes with distinct microbial and functional profiles.

**Methods:**

In a cohort of 118 PLWH, we analyzed the gut microbiome in relation to weight gain and ART regimen using *16S rRNA* gene sequencing, taking enterotype classification into account.

**Results:**

The enterotype was strongly associated with sexual orientation. Of the 67 individuals forming a *Prevotella*-dominated enterotype cluster in principal coordinates analysis, 93% were men who had sex with men (MSM), while 31% of individuals in the *Bacteroides*-dominated enterotype cluster were MSM and 69% were non-MSM. Forty-nine genera differed significantly between the MSM and non-MSM individuals. When stratified by dominant genus, only six taxa were associated with weight gain. Of these, five were restricted to *Bacteroides*-dominated individuals. Among them, the class Actinobacteria and genus *Bifidobacterium* differed between individuals gaining more than 5% weight and less than 5% weight 1 year after ART switch. Additionally, three taxa were significantly different between 15% of individuals with the highest weight gain (≥6.3%) and the highest weight loss (≤3.19%) 1 year after ART switch, including the phyla Firmicutes, Verrucomicrobia, and Synergistetes. Distinct functional properties in *Bacteroides*, but not *Prevotella*-dominated enterotype individuals, linked to weight gain were observed, particularly for glycan and lipid metabolism. Additionally, ART regimen-associated differences were observed for the phylum Actinobacteria, although this was limited to *Prevotella-*dominated enterotype individuals.

**Discussion:**

Differences in the composition and functional characteristics of the gut microbiome associated with weight gain and ART regimens were enterotype-specific and relatively small compared with differences linked to sexual orientation. Due to the substantial differences in gut microbiome structure among many MSM, categorization into enterotypes is useful for identifying differences in microbiome composition associated with variables such as weight gain or ART, which may be limited to a single enterotype. This may further advance the identification of microbes that contribute to weight gain or alter the gut microbiome composition in the context of the enterotype.

## 1 Introduction

Advances in the management and early initiation of antiretroviral therapy (ART) have improved the life expectancy and quality of life of people living with HIV (PLWH) in recent decades. However, ART is associated with adverse side effects including weight gain and metabolic dysfunction (Bailin et al., 2020; Mac Cann et al., 2024). The two integrase strand transfer inhibitors dolutegravir (DTG), bictegravir (BIC), and the nucleotide analog reverse transcriptase inhibitor tenofovir alafenamide are associated with an increased likelihood of excess weight gain in PLWH (Venter et al., 2019; Sax et al., 2020; Erlandson et al., 2021; Sokhela et al., 2024). However, the cause and pathogenesis of weight gain during ART in PLWH are not well understood.

Gut microbiota contributes to the metabolism of food components and is involved in weight gain and obesity. Animal studies have revealed that the gut microbiota influences energy harvesting from available food components (Turnbaugh et al., 2006), extent of food consumption and food selection behavior (Vijay-Kumar et al., 2010; Trevelline and Kohl, 2022), and regulation of dietary polysaccharide digestion, lipid absorption, metabolism, and storage (Backhed et al., 2004; Flint et al., 2008; Wang et al., 2023). In humans, several characteristic features of the gut microbiota have been described to classify individuals as lean or obese (Le Chatelier et al., 2013; Walters et al., 2014); however, these findings have not been consistently observed across different studies conducted worldwide. One possible reason for the inhomogeneous findings in humans is the fundamental differences in gut microbiota composition between healthy individuals. Gut microbiota can be classified into specific community structures, the so-called enterotypes, which are based on an indicator taxon, defined as the genus with the highest relative abundance, and the microorganisms that typically co-occur with this indicator taxon (Arumugam et al., 2011). The two major enterotypes are the *Bacteroides* enterotype, the most prevalent enterotype in Westernized populations, and the *Prevotella* enterotype, which is typically underrepresented in Westernized countries and the most prevalent enterotype in non-Western countries, where inhabitants follow a traditional lifestyle and consume diets rich in unprocessed foods (Arumugam et al., 2011; Costea et al., 2018). Enterotypes are considered stable over long periods, have distinct digestive functions, and show different responses to weight changes in specific diets (Christensen et al., 2018). The metabolic response to foods differs among individuals depending on the gut microbiota composition (Zeevi et al., 2015; Zmora et al., 2016), suggesting that the gut microbiota plays a role in obesity treatment.

Beyond the known factors that influence gut microbiome composition, including diet, lifestyle, diseases, and drugs, much of the composition of gut microbiota remains unknown. The vast majority of men who have sex with men (MSM) have distinctive gut microbiota, with *Prevotella* being the predominant genus (Noguera-Julian et al., 2016; Armstrong et al., 2018; Kehrmann et al., 2019). Notably, the gut microbiota specificities in MSM are independent of HIV infection (Noguera-Julian et al., 2016; Armstrong et al., 2018; Tuddenham et al., 2020). We have recently shown that the MSM gut microbiome shares many features with the gut microbiome of non-Westernized populations, is distinct from the typical *Bacteroides*-dominated gut microbiome of the majority of individuals in Westernized populations, and is independent of known factors that influence gut microbiota composition, including diet and lifestyle (Huang et al., 2024). In the present study, we analyzed the gut microbiome in PLWH and compared the differences in the gut microbiome composition associated with sexual orientation with those linked to weight gain and ART, with consideration of the enterotype.

## 2 Materials and methods

### 2.1 Study population

The study was reviewed and approved by the Ethics Committee of the Medical Faculty of the University of Duisburg-Essen (20-9159-BO) and was performed in accordance with the latest version of the Declaration of Helsinki. Written informed consent was obtained from all patients before enrolment. All participants were part of the HIV-HEART cohort, a long-term prospective, multicenter observational study cohort in the German Ruhr area, which analyses the relevance of HIV infection on the incidence, prevalence, and progression of coronary heart disease in PLWH since 2004 (Neumann et al., 2007). We recruited 130 PLWH, and of these, we received stool samples from 118 individuals that were analyzed in this study.

### 2.2 Sample processing

Stool samples were collected in stool collection tubes filled with liquid DNA stabilization buffer (Invitek, Berlin, Germany) and stored at −80°C until DNA extraction. DNA was extracted using the PSP^®^ Spin Stool DNA Basic Kit (Invitek) according to the protocol for stool samples with “difficult-to-lyse bacteria.” Amplicon and index PCRs, and library preparation were performed as previously described (Kehrmann et al., 2019). Sequencing was performed on an Illumina MiSeq using the Illumina MiSeq 600 Cycle Reagent Kit 3. Fastq sequences and metadata were deposited in the National Center for Biotechnology Information repository SRA (sequencing read archive) under Bioproject accession number PRJNA1208265.

### 2.3 Microbiome data analysis

We demultiplexed fastq-files of forward and reverse sequences per sample using the QIIME2 (Quantitative Insights Into Microbial Ecology2) pipeline (Bolyen et al., 2019) with the DADA2-package. Chimeric sequences were filtered using the consensus method. We obtained 1,203,899 quality-filtered sequences from 118 samples of 118 PLWH with a minimum of 5,667 and a maximum of 24,358 quality-filtered sequences per sample and a mean/median number of 10,202/10,080 sequences per sample. Diversity metrics were calculated using a rarefaction depth of 5,667 sequences. The q2-feature-classifier plugin was used for taxonomy assignment with a Naïve Bayes classifier, trained on the Greengenes 13_8_99% OTUs *16S rRNA* gene full-length sequences. The Kruskal–Wallis test, performed with the QIIME diversity alpha-group-significance command, was applied for comparisons between categorical metadata columns and alpha diversity metrics. Differences in beta diversity among groups were calculated using the QIIME diversity beta-group significance command with permutational multivariate analysis of variance (PERMANOVA) of distance matrices with 999 permutations. Singletons were filtered from the BIOM-tables before performing differential abundance analyses were performed. For association analyses of the two groups with microbial taxa, we performed MaAsLin2 analysis (Mallick et al., 2021) using Galaxy lab on non-transformed relative abundances with the default filtering parameters of a minimum prevalence of 0.1 for which a feature was detected at minimum abundance, and a q-value threshold of 0.25. The default MaAsLin2 Benjamini–Hochberg procedure was used to control the False Discovery Rate (FDR).

Microbial functions were predicted using PICRUSt2 (version 2.5.3) (Douglas et al., 2020), with default parameters. All samples met the minimum quality threshold of 1,500 total frequencies, and sequences with frequencies <15 were excluded. The predicted KEGG Orthology (KO) abundances per sample were mapped to the Kyoto Encyclopedia of Genes and Genomes (KEGG) pathways by grouping KO numbers into their respective pathways and summing their abundances. The resulting values were assigned to the corresponding pathways to generate pathway-level abundance. Differential pathway abundance analysis was performed using ALDEx2 (version 1.34.0) (Fernandes et al., 2014) with default parameters and 1,000 Monte Carlo simulations. Pathways were considered significantly differentiated if they met the following criteria: (1) a Benjamini–Hochberg corrected p-value from the Wilcoxon Rank Sum test below 0.05, and (2) a minimum median difference of 0.58 in log-centered abundance values between groups. Differentiated pathways were annotated based on their names and class information from the KEGG database. The full pipeline and analysis scripts for the functional microbiome analysis are publicly available at: https://github.com/AlirezaDostmohammadi/HIV-weight.

We tested for significant differences in patient characteristics using ordinary one-way ANOVA for metric variables and the chi-square test for categorical variables with a significance level <.05. Alpha diversity metrics, bar plots, boxplots, and principal coordinates analysis (PCoA) were visualized using Dokdo Version 1.15 (https://github.com/sbslee/dokdo).

## 3 Results

### 3.1 Clinical characteristics of the study population

Stool samples were obtained from 118 PLWH, of which 106 were of white ethnicity (90%), 95 (80%) were male (79 MSM), and 23 (20%) were female (**Table 1**). The HIV viral load was <50 copies/ml in 117 of the 118 participants. The mean age of the cohort was 55.1 ± 11.0 years, with a mean duration of HIV infection of 16.7 ± 8.7 years. The nutritional status at the time of stool sampling, according to the World Health Organization (WHO) classification of body mass index (BMI), was normal in 45 participants, pre-obesity in 47, and obesity in 26, including 20 classified as obesity class I and 3 as class II and III, respectively. The antiretroviral therapy (ART) regimen included DTG in 36 participants, BIC in 31 participants, tenofovir alafenamide without BIC (TAF) in 40 participants, and 11 participants who received other ART regimens. Stool samples for microbiome analyses were collected after 3.9 ± 2.4 years of the current ART regimen.

**Table 1:** Characteristics of PLWH participating in the study.

Characteristic	Total	DTG	BIC	TAF	other	*P-*value
No. of subjects	118	36	31	40	11	
Age, y, mean ± SD	55.1 ± 11.0	57.6 ± 11.0	53.1 ± 9.3	54.2 ± 11.3	56.0 ± 14.3	0.31
Sex						
male	95 (80.5)	31 (86.1)	27 (87.1)	31 (77.5)	6 (54.5)	0.09
female	23 (19.5)	5 (13.9)	4 (12.9)	9 (22.5)	5 (45.5)	
ISCED score, mean ± SD	4.1 ± 1.1	4.0 ± 1.1	4.4 ± 1.2	4.2 ± 0.9	3.4 ± 0.7	0.16
BMI, kg/m^2^, mean ± SD	26.9 ± 4.7	27.9 ± 5.5	26.4 ± 4.6	26.7 ± 4.2	26.0 ± 4.4	0.45
Duration of HIV infection, y, mean ± SD	16.7 ± 8.7	18.0 ± 7.9	13.0 ± 8.7	17.9 ± 8.7	18.4 ± 9.2	0.80
CDC HIV stage-clinical						
A	51 (43.2)	14 (38.9)	16 (51.6)	17 (42.5)	4 (36.4)	0.07
B	33 (28.0)	14 (38.9)	9 (29.0)	10 (25.0)	0 (0.0)	
C (AIDS)	34 (28.8)	8 (22.2)	6 (19.4)	13 (32.5)	7 (63.6)	
CDC HIV stage-immunological						
I	14 (11.9)	3 (8.3)	3 (9.7)	6 (15.0)	2 (18.2)	0.85
II	50 (42.4)	15 (41.7)	16 (51.6)	15 (37.5)	4 (36.4)	
III	54 (45.8)	18 (50.0)	12 (38.7)	19 (47.5)	5 (45.5)	
Ratio CD4/CD8 cells, mean ± SD	0.95 ± 0.46	0.89 ± 0.47	0.99 ± 0.48	0.95 ± 0.44	1.02 ± 0.46	0.95
HIV RNA <50 copies/ml	117 (99.2)	36 (100.0)	31 (100.0)	39 (97.5)	11 (100.0)	0.58
Sexual behavior						
MSM	79 (66.9)	29 (80.6)	20 (64.5)	25 (62.5)	5 (45.5)	0.12
nonMSM	39 (33.1)	7 (19.4)	11 (35.5)	15 (37.5)	6 (54.5)	
Diabetes mellitus	14 (11.9)	7 (19.4)	3 (9.7)	3 (7.5)	1 (9.1)	0.40
Hyperlipidemia	66 (55.9)	25 (69.4)	13 (41.9)	21 (52.5)	7 (63.6)	0.13
Arterial hypertension	48 (40.7)	20 (55.6)	10 (32.3)	12 (30.0)	6 (54.5)	0.07
Lipodystrophy	15 (12.7)	8 (22.2)	0 (0.0)	3 (7.5)	4 (36.4)	0.003
Duration of actual ART, y						
Mean ± SD	3.9 ± 2.4	4.3 ± 1.8	2.4 ± 1.1	3.9 ± 1.5	7.0 ± 4.9	<0.001
Median (IQR)	3 (2-5)	5 (3-6)	2 (2-3)	4 (3-5)	7 (2-12)	
History of COPD	6 (5.1)	2 (5.6)	1 (3.2)	1 (2.5)	2 (18.2)	0.20
History of CMV infection	1 (0.8)	0 (0.0)	0 (0.0)	1 (2.5)	0 (0.0)	0.58
History of hepatitis B	3 (2.5)	1 (2.8)	1 (3.2)	1 (2.5)	0 (0.0)	0.95
History of hepatitis C	9 (7.6)	3 (8.3)	1 (3.2)	3 (7.5)	2 (18.2)	0.45
History of depression	25 (21.2)	10 (27.8)	6 (19.4)	6 (15.0)	3 (27.3)	0.54
Use of antibiotics within 6 months before stool sampling	4 (3.4)	0 (0.0)	2 (6.5)	2 (5.0)	0 (0.0)	0.39
Ethnicity						
White	106 (89.8)	34 (94.4)	27 (87.1)	35 (87.5)	10 (90.9)	0.72
Other	12 (10.2)	2 (5.6)	4 (12.9)	5 (12.5)	1 (9.1)	
Weight gain 1 year after ART change [%]						
Median (IQR)	1.32 (-1.21-3.91)	1.47 (-1.09-5.22)	1.18 (-1.21-3.66)	1.59 (-1.18-4.90)	-0.58 (-2.20-10.74)	0.49
More 5	24 (20.3)	9 (25.0)	3 (9.7)	9 (22.5)	3 (27.3)	0.36
Less 5	93 (78.8)	26 (72.2)	28 (90.3)	31 (77.5)	8 (72.7)	
Diet	4 (3.4)	1 (2.8)	0 (0.0)	2 (5.0)	1 (9.1)	0.54

Data are presented as No. (%) unless otherwise indicated. Metric variables were tested with ordinary one-way ANOVA. Categorical variables were compared by chi-square test. Mean ± SD was used for metric variables. For skewed, approximately log-normal distributions, medians and IQR are reported.

ART, antiretroviral therapy; BIC, bictegravir; BMI, body mass index; CDC, Centers for Disease Control and Prevention; CMV, cytomegalovirus; COPD, chronic obstructive pulmonary disease; DTG, dolutegravir; IQR, interquartile range; ISCED, International Standard Classification of Education; MSM, men who have sex with men; SD, standard deviation; TAF, tenofovir alafenamide without BIC.

### 3.2 The majority of MSM gut microbiomes is significantly distinct from the gut microbiomes of non-MSM individuals and characterized by a *Prevotella* dominated gut microbiota, which exhibits distinct functional properties

**Figure 1:**
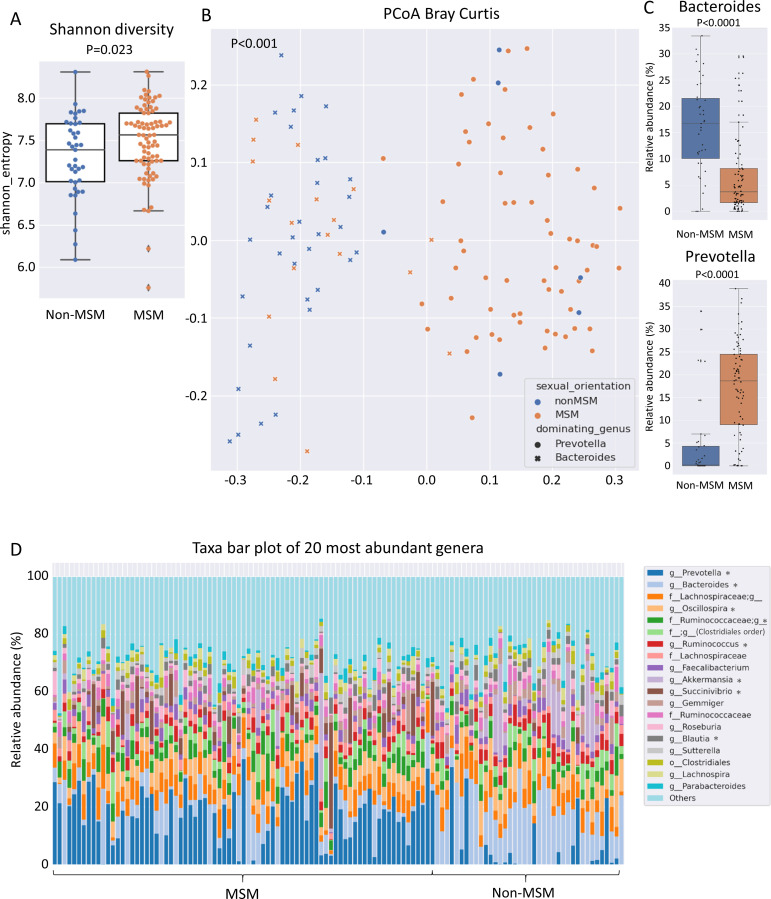
Alpha and beta diversity of the gut microbiome of PLWH linked to sexual behavior. **(A)** Shannon diversity index, **(B)** principal coordinates analysis and **(C)** relative abundance of the genera *Bacteroides* and *Prevotella*, in individual gut microbiome samples from men who have sex with men (MSM) represented in orange and non-MSM represented in blue. **(D)** Taxa bar plot of the 20 most abundant genera in the MSM and non-MSM individuals. f__;g__represents the OTUs that matched the Greengenes reference database but could not be assigned below the order level.;g__represents operational taxonomic unit that matched the Greengenes database and could not be assigned below the family level. Asterisks in the figure legend indicate genera that were identified as significantly different by MaAsLin2 analysis with a q value <0.25 between MSM and non-MSM individuals. The q value illustrates the corrected significance reported by MaAsLin2 computed using the Benjamini–Hochberg method. PLWH, people living with HIV; MSM, men who have sex with men.

**Figure 2:**
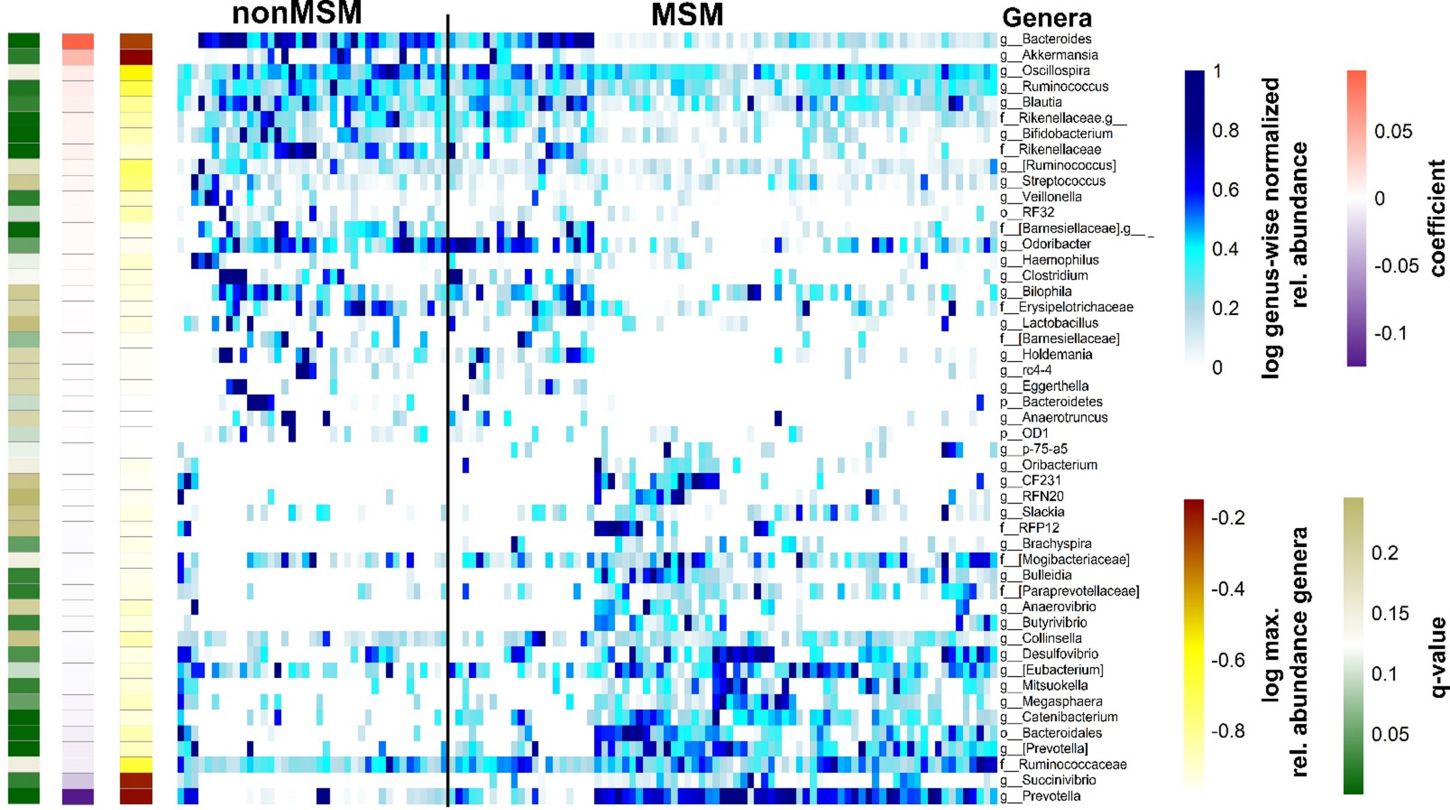
Gut microbiome genera of PLWH associated with sexual behavior. Heatmap of genera in individual samples identified as significantly different by MaAsLin2 between men who have sex with men (MSM) and non-MSM, ordered by sexual orientation. The blue heatmap shows normalized relative abundance data, which were log2 transformed. A genus wise minimum-maximum normalization was performed to standardize the value ranges for better comparability across the samples. The black vertical line indicates distinctions between the *Bacteroides-* and *Prevotella*-dominated groups. The single-column heatmaps provide additional information about differentiated genera for each condition. The logarithmic maximum relative abundance of each genus is visualized by light yellow (minimum) to red color (maximum). The effect size reported by the MaAsLin2 as model coefficient is shown in purple to dark orange color. The q value illustrates the corrected significance reported by MaAsLin2 computed with the Benjamini–Hochberg method. PLWH, people living with HIV; MSM, men who have sex with men.

**Figure 3:**
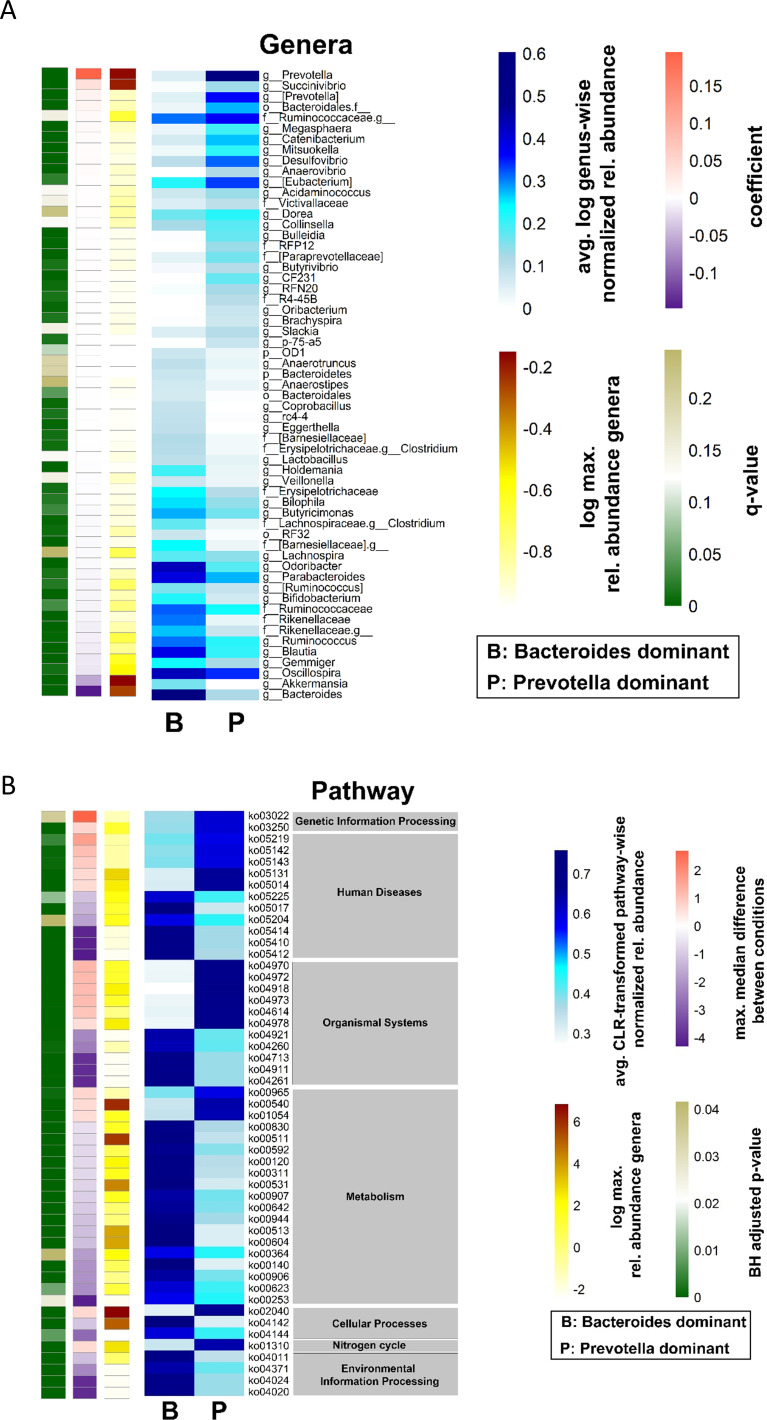
Differences in bacterial genera and predicted gut microbiome functions between *Prevotella* and *Bacteroides* dominated gut microbiome samples from PLWH. **(A)** The heatmap shows the group average abundance of significantly different abundant genera in *Bacteroides* and *Prevotella* dominated gut microbiome samples, which were identified by MaAsLin2. The blue heatmap shows the normalized relative abundance data, which was log2 transformed. A genus wise minimum-maximum normalization was performed to standardize value ranges for better comparability across samples. The single-column heatmaps provide additional information about differentiated genera for each condition. The logarithmic maximum relative abundance of each genus is visualized by light yellow (minimum) to red color (maximum). The effect size reported by the MaAsLin2 as model coefficient is shown in purple to dark orange. The q value illustrates the corrected significance reported by MaAsLin2 computed with the Benjamini–Hochberg method. **(B)** Heatmap of the group average of significantly different KEGG orthologs and their functional pathways and effect size of gut microbiome samples of *Prevotella-* and *Bacteroides*-dominated gut microbiomes. A pathway-wise ranked normalization was performed. The relative pathway abundances were calculated from the PICRUSt2 output, and the centered-log ratio transformation was performed. The blue heatmap shows the centered-log-transformed pathway-wise normalized relative abundance from PICRUSt2. ALDEx2 was used to compute the statistical differences between the two groups. The single-column heatmaps provide additional information about differentiated genera for each condition. The logarithmic maximum relative abundance of each genus is visualized by light yellow (minimum) to red color (maximum). The maximum median difference between the conditions is shown in purple to dark orange. The adjusted p-value was computed using the Benjamini–Hochberg method and visualized in green. PLWH, people living with HIV; MSM, men who have sex with men.

Many MSM exhibit a specific gut microbiome that is characterized by high alpha diversity metrics and a high *Prevotellaceae* abundance (Noguera-Julian et al., 2016; Armstrong et al., 2018; Kehrmann et al., 2019). The Shannon diversity index of the gut microbiome in the present cohort was higher in MSM than in non-MSM individuals (p = 0.023, **Figure 1A**). Multivariate analysis (PERMANOVA) of unweighted and weighted UniFrac, Bray–Curtis, and Jaccard distance matrices revealed that the gut microbiome was significantly different between MSM and non-MSM individuals (p <0.001 for all distance matrices; **Figure 1B** and **Supplementary Figure 1**). Principal coordinate analysis (PCoA) of our PLWH cohort separated the gut microbiome samples into two clusters. The vast majority (92.6%) of one cluster comprised MSM samples (63 MSM of 68 individuals in this cluster), and the other cluster comprised 16 MSM (31.3%) and 34 non-MSM individuals. *Prevotella* in MSM and *Bacteroides* in non-MSM gut microbiota samples were the genera with the highest relative abundance, serving as drivers of the separation of the two clusters: *Prevotella*-dominated samples with a P/B ratio>1 or *Bacteroides*-dominated samples with a P/B ratio <1 (**Figures 1B, C**). Eight of the 20 most abundant genera of the gut microbiome showed significantly different in abundance between MSM and non-MSM (**Figure 1D**). In total, MaAsLin2 identified 49 genera that were significantly different in abundance between the MSM and non-MSM groups (**Figure 2**), whereas 59 genera differed between *Prevotella-* and *Bacteroides*-dominated enterotypes in this study (**Figure 3A**). Interestingly, nearly half of the 59 genera that significantly differed between *Prevotella-* and *Bacteroides*-dominated enterotype individuals in this study were also among the 51 significantly different genera identified in a previous study that investigated the gut microbiome of PLWH. The genera *Bacteroides*, *Akkermansia*, *Blautia*, *Parabacteroides*, *Clostridium*, *Holdemania*, *Eggerthella*, *Coprobacillus*, and *Anaerostipes* were overrepresented in *Bacteroides*-dominated enterotype individuals and the genera *Prevotella*, *Succinivibrio*, [*Prevotella*], *Megasphaera*, *Catenibacterium*, *Mitsuokella*, *Desulfovibrio*, *Anaerovibrio*, [*Eubacterium*], *Bulleidia*, *Butyrivibrio*, the genus CF231 of [*Paraprevotellaceae*], the genus RFN20 of *Erysipelotrichaceae*, *Brachyspira*, *Slackia*, the genus p-75-a5 of *Erysipelotrichaceae* were overrepresented in the *Prevotella* dominated gut microbiomes of both cohorts (**Supplementary Table 1**). In addition, four genera were significantly different between *Prevotella-* and *Bacteroides*-dominated enterotypes in both cohorts with the same taxonomic assignment; however, the taxonomy could only be assigned above the genus level (**Supplementary Table 1**).

PICRUSt2 identified differences in the functional properties of *Bacteroides-* and *Prevotella*-dominated gut microbiota samples. A total of 51 KEGG pathways were significantly different between the two groups. The highest number of different KEGG pathways (19) was related to metabolic functions, of which 16 were overrepresented in the *Bacteroides*-dominated group and three were overrepresented in the *Prevotella*-dominated gut microbiome. Eight pathways were found to be involved in glycan biosynthesis, metabolism, and lipid metabolism (**Figure 3B**; **Supplementary Table 2**).

### 3.3 The differences in the gut microbiome composition associated with weight gain are linked to the *Bacteroides* enterotype

**Figure 4:**
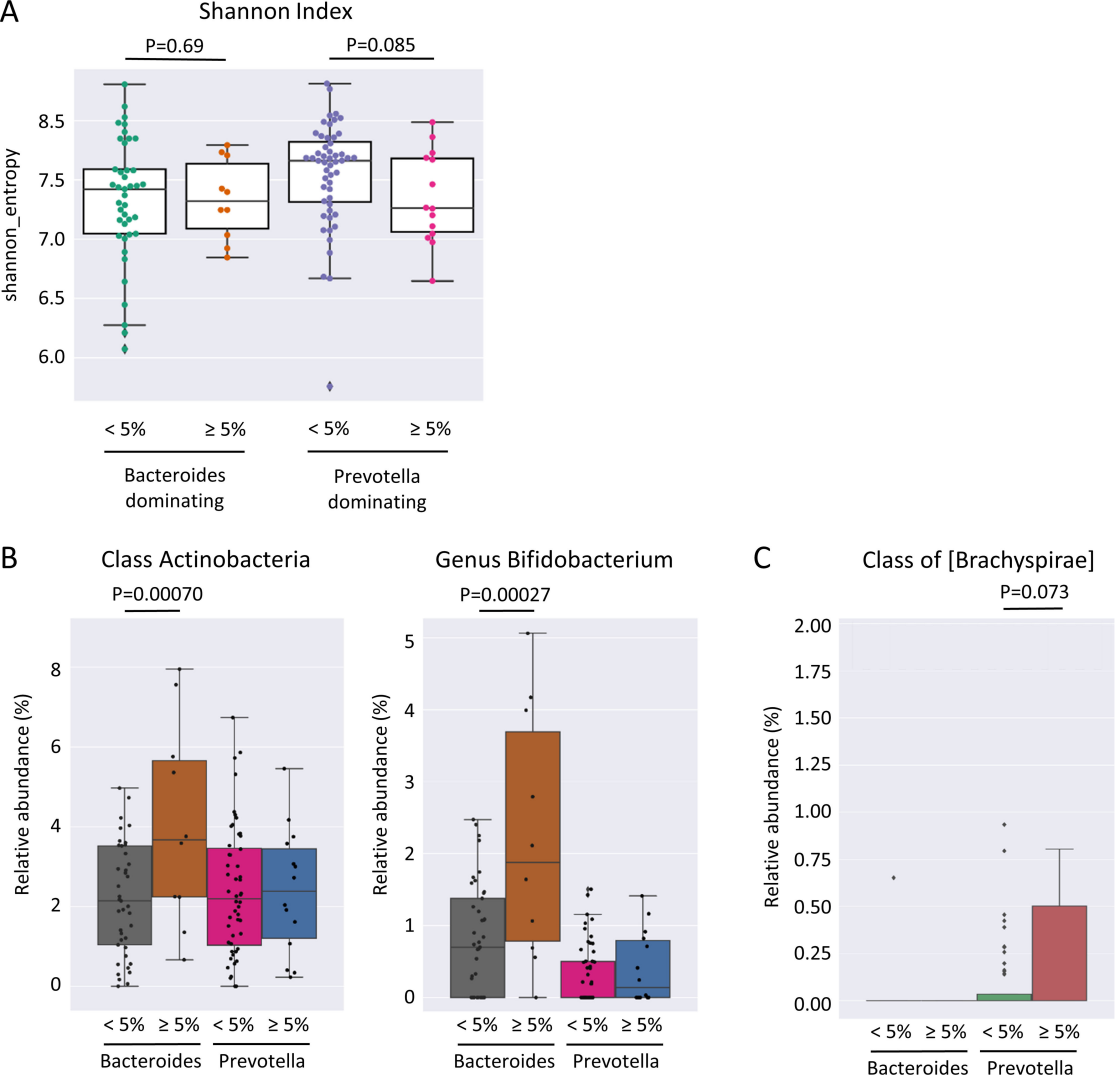
Differences in the gut microbiome of PLWH associated with weight gain 12 months after ART switch. **(A)** Shannon diversity index of individuals gaining more or less than 5% weight 12 months after antiretroviral therapy (ART) switch separated by the enterotype. **(B)** Relative abundance of class Actinobacteria and genus *Bifidobacterium* and **(C)** class [Brachyspirae] in individuals gaining more or less than 5% weight 12 months after ART change separated by the enterotype. PLWH, people living with HIV; ART, antiretroviral therapy.

**Figure 5:**
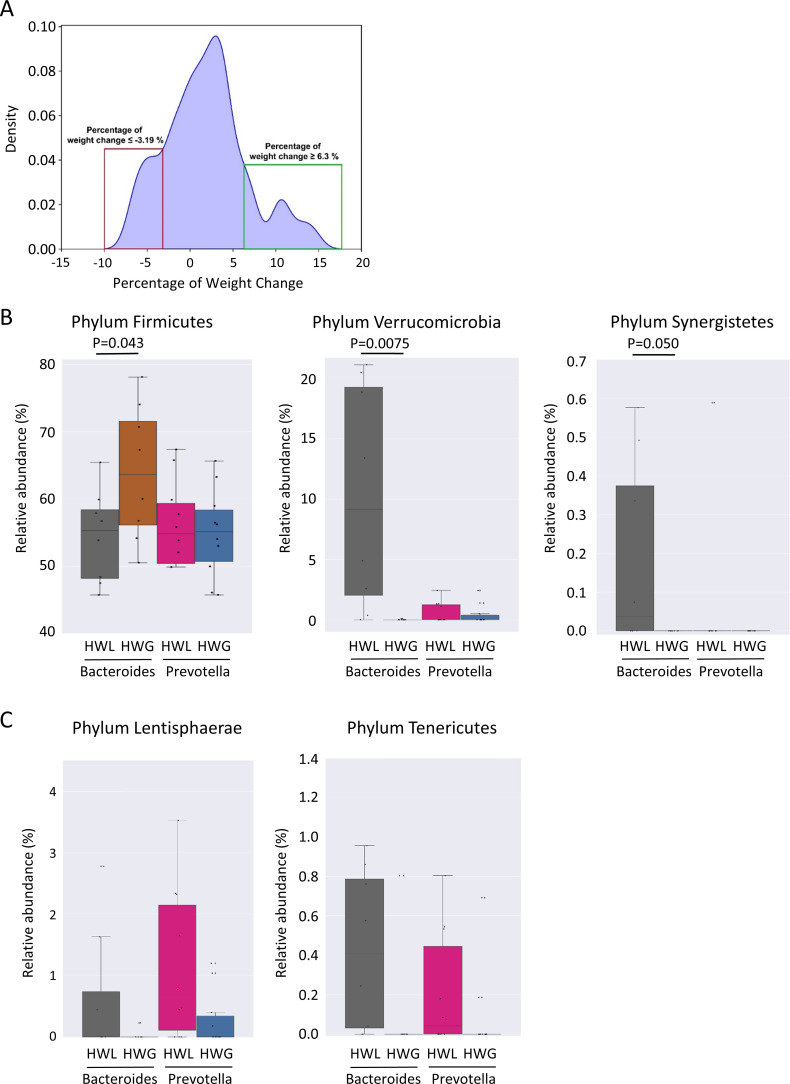
Differences in the gut microbiome of PLWH associated with weight gain in the 15% of individuals at both extremes of the weight change distribution. **(A)** Distribution of percentage of weight change 12 months after antiretroviral therapy (ART) change in individuals participating in the study. Individuals with a weight change of ≥6.3% and ≤−3.19% 12 months after ART switching, representing the 15% of individuals at both extremes of the weight change distribution, are classified as having high weight loss (HWL) or high weight gain (HWG). **(B)** Relative abundance of the phyla Firmicutes, Verrucomicrobia, and Synergistetes **(B, C)** the phyla Lentisphaerae and Tenericutes in the HWL and HWG groups. PLWH, people living with HIV; ART, antiretroviral therapy; HWL, high weight loss; HWG, high weight gain.

**Figure 6:**
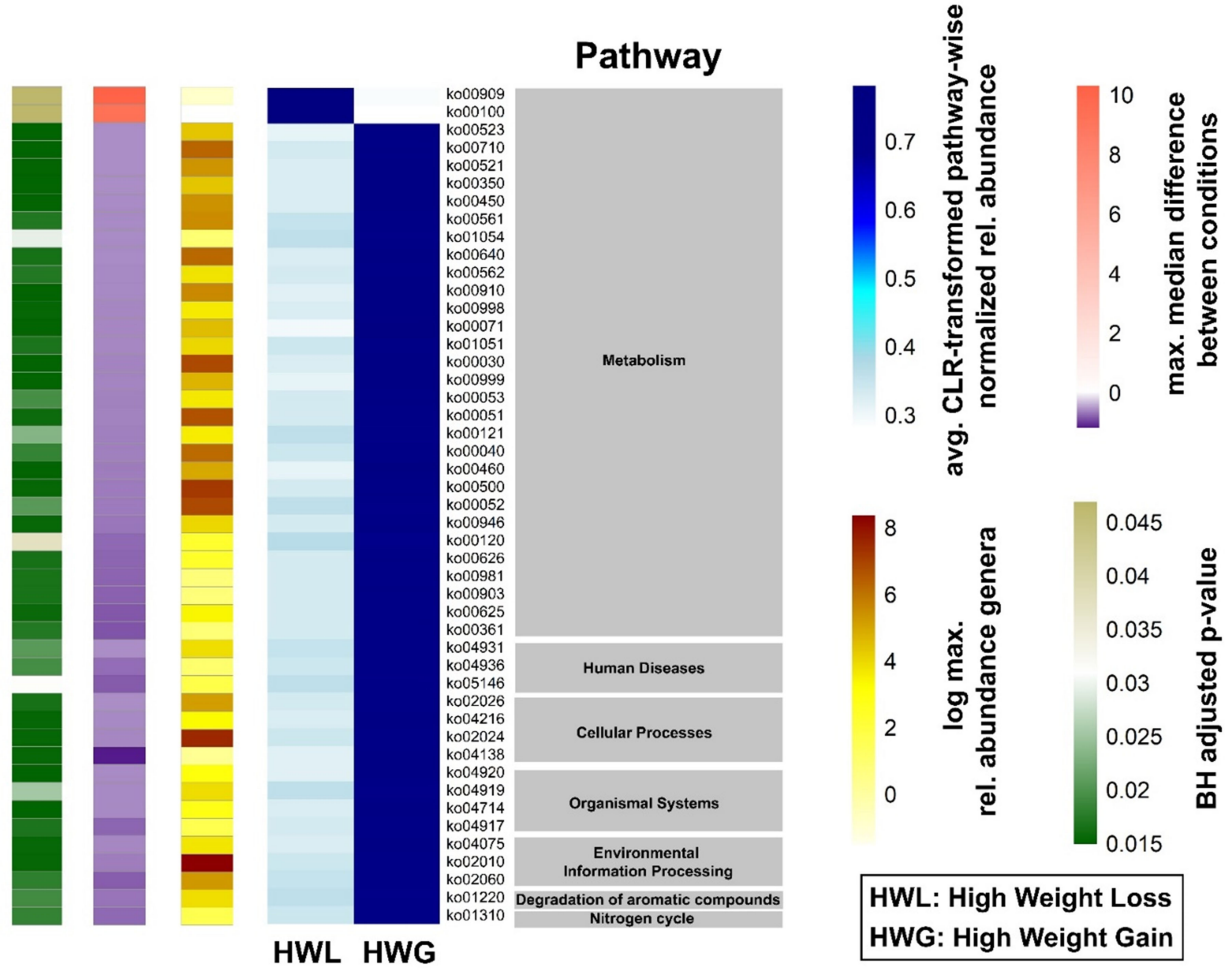
Differences in the predicted gut microbiome functions associated with weight gain in the 15% of *Bacteroides* enterotype individuals at both extremes of the weight change distribution. Heatmap of group average of significantly different KEGG orthologs and their functional pathways and effect size of *Bacteroides* enterotype gut microbiomes of HWL and HWG groups. The relative pathway abundances were calculated from PICRUSt2 output and centered-log ratio transformation was performed. The blue heatmap shows the average centered log-ratio transformed pathway-wise normalized relative abundance for each pathway. ALDEx2 was used to compute statistical differences between the two groups. The single-column heatmaps provide additional information about differentiated genera for each condition. The logarithmic maximum relative abundance of each genus is shown by light yellow (minimum) to red (maximum). The maximum median difference between the conditions is visualized in purple to dark orange. The adjusted p-value was computed with the Benjamini–Hochberg method and shown in green. HWL, high weight loss; HWG, high weight gain.

No significant differences in Shannon diversity and richness were found in individuals who gained more than 5% weight 12 months after ART switching without consideration of the enterotype (p = 0.15). The *Prevotella*-dominated gut microbiota of PLWH who gained more than 5% of their body weight one year after ART change was characterized by a tendency towards a lower Shannon diversity compared to those who gained less than 5% weight in the same period (p = 0.085) (**Figure 4A**). In contrast, individuals with a *Bacteroides*-dominated gut microbiota demonstrated comparable Shannon diversity between those gaining more than 5% of their body weight and those who gained less weight 12 months after ART switch (p = 0.69). The class Actinobacteria (p = 0.00070) and genus *Bifidobacterium* (p = 0.00027) were the only taxa associated with weight gain exceeding 5% within 12 months after ART change when considering all individuals with a *Bacteroides*-dominated gut microbiome (**Figure 4B**). The relative abundance of Actinobacteria and *Bifidobacterium* was significantly increased only in individuals with a *Bacteroides*-dominated microbiome, whereas the increase observed in individuals with a *Prevotella*-dominated gut microbiome was not statistically significant (**Figure 4B**). When only considering individuals with a *Prevotella* enterotype, the low-abundant family [*Brachyspirae*] was more abundant in individuals who gained more than 5% weight 12 months after ART change (**Figure 4C**). In addition, we analyzed differences in gut microbiota composition and functional characteristics between the two tails of the 15th and 85th percentiles of individuals who gained weight by comparing the differences in gut microbiome composition between individuals who lost ≤-3.19% (HWL) and those who gained ≥6.3% weight (HWG) (**Figure 5A**). At the phylum level, the relative abundance of Firmicutes was significantly higher and that of Verrucomicrobia and Synergistetes was lower in *Bacteroides* enterotype individuals with the highest weight gain compared to those with the highest weight loss, while the differences for *Prevotella* enterotype individuals at the phylum and genus levels were insignificant (**Figure 5B**). A trend towards higher relative abundance in individuals with high weight loss compared to those with high weight gain 12 months after ART switching was observed for the phyla Lentisphaerae and Tenericutes for individuals of both enterotypes (**Figure 5C**). A total of 47 KEGG pathways significantly differed between the HWG and HWL individuals in *Bacteroides* enterotype individuals, of which 31 were metabolic pathways, including eight carbohydrate, five lipid, three amino acid, and two energy metabolism pathways. Sixteen pathways were other functions, including human diseases (3), cellular processes (4), organismal systems (4), environmental information processing (3), degradation of aromatic compounds (1), and the nitrogen cycle pathway (1) (**Figure 6**; **Supplementary Table 3**). No single KEGG pathway was significantly different between HWG and HWL individuals with the *Prevotella* enterotype (**Supplementary Table 4**).

### 3.4 Most differences in the gut microbiota composition linked to ART regimens are enterotype specific

**Figure 7:**
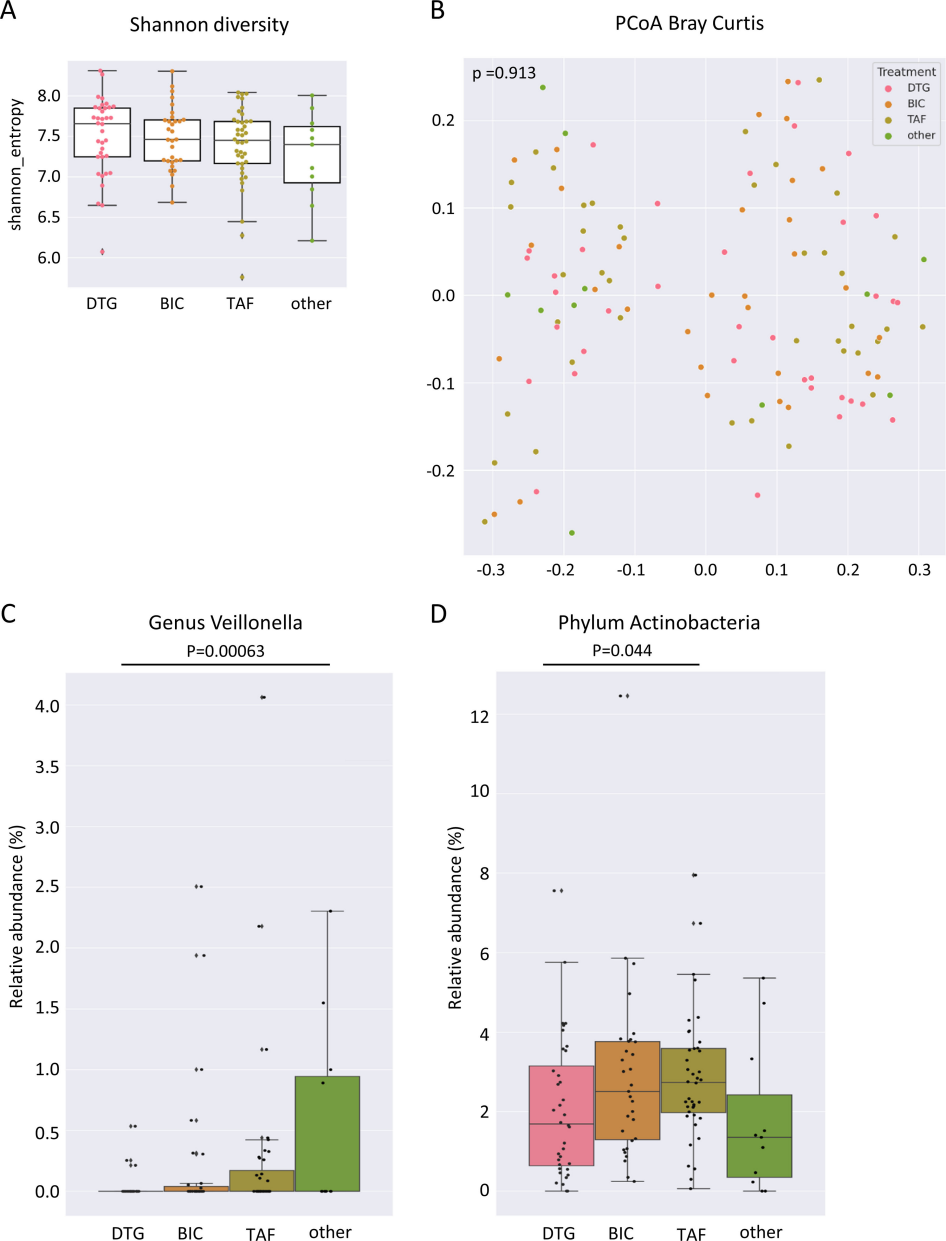
Gut microbiome associated with ART regimen in PLWH. **(A)** Shannon diversity index of gut microbiome samples of people living with HIV (PLWH) treated with dolutegravir (DTG), bictegravir (BIC), tenofovir alafenamide without BIC (TAF), or other antiretroviral regimens (other ART). **(B)** Principal coordinates analysis of Bray–Curtis distance matrix of individual gut microbiome samples treated with DTG-, BIC-, TAF containing and other ART regimens. **(C)** Relative abundance of the genus *Veillonella* and **(D)** the phylum Actinobacteria in individual gut microbiome samples treated with DTG-, BIC-, TAF without BIC-containing and other ART regimens. PLWH, people living with HIV; DTG, dolutegravir; BIC, bictegravir; TAF, tenofovir alafenamide regimen without BIC; other ART, other antiretroviral regimens.

Various drugs have been shown to influence the structure of gut microbiota. However, no relevant differences were observed for the gut microbiota in Shannon diversity and PERMANOVA multivariate analysis for Bray–Curtis distance matrix regarding two-group comparisons between DTG, BIC, TAF, or other ART-containing regimens in our study when considering all individuals (**Figures 7A, B**).

**Figure 8:**
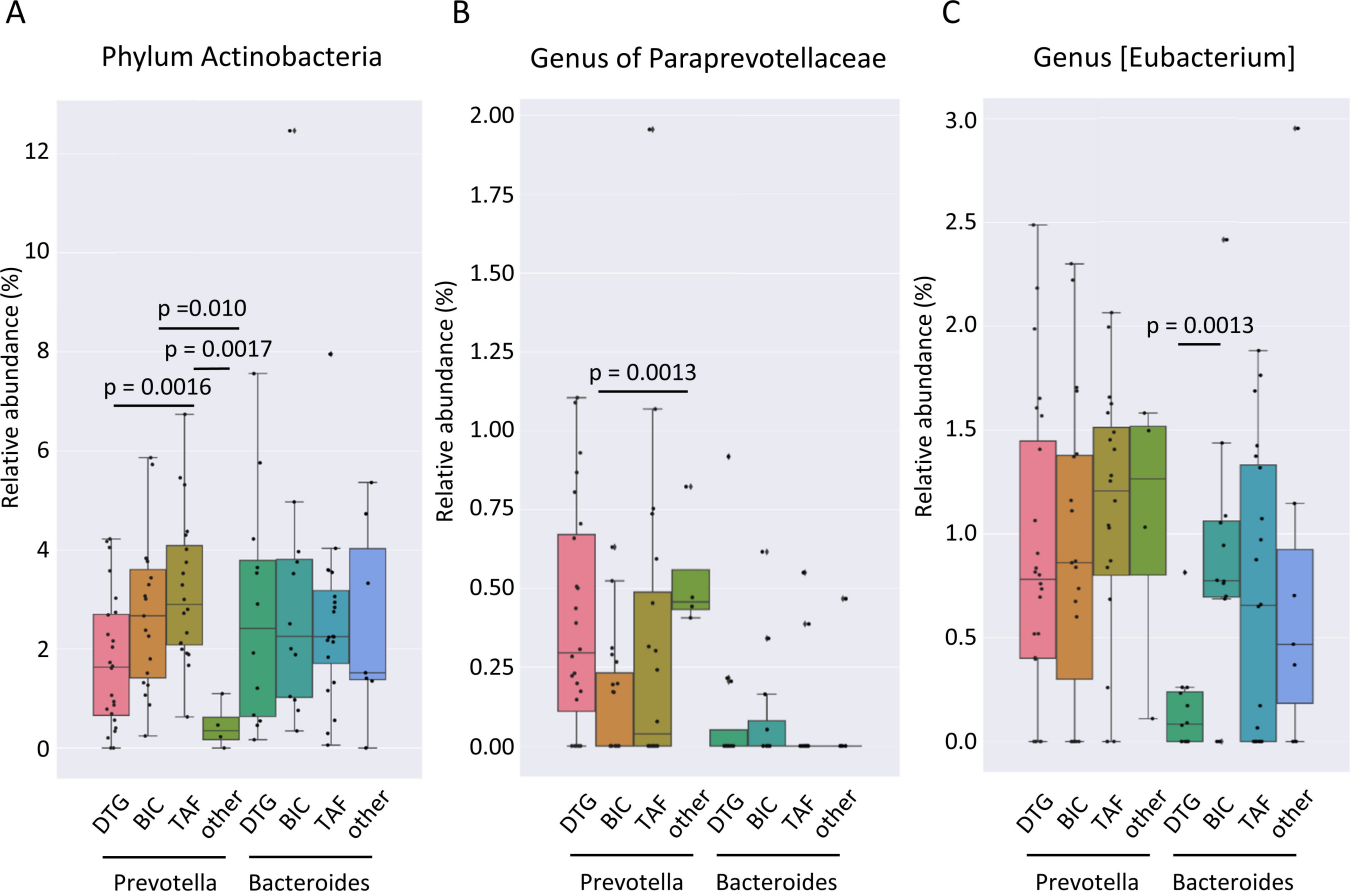
Gut microbiome associated with ART regimen in PLWH stratified by the dominating genus. **(A)** Relative abundance of the gut microbiome of Actinobacteria phylum, **(B)** a genus of *Paraprevotellaceae*, and **(C)** genus [*Eubacterium*] in different antiretroviral regimens in gut microbiomes stratified by the dominating genus of the enterotype. PLWH, people living with HIV; DTG, dolutegravir; BIC, bictegravir; TAF, tenofovir alafenamide regimen without BIC; other ART, other antiretroviral regimen.

The only genus identified as significantly different by MaAsLin2 between all individuals receiving DTG and those receiving other ART-containing regimens was *Veillonella* (**Figure 7C**). No additional genera were identified as significantly different between the treatment regimens when all possible combinations of the two treatments were compared. At the phylum level, the relative abundance of Actinobacteria was higher in the gut microbiome of TAF treated compared to DTG-treated individuals when all individuals were considered (**Figure 7D**). A comparison of the gut microbiome composition according to enterotype revealed notable differences in the phylum Actinobacteria between the various treatment groups of individuals with a *Prevotella* enterotype. The TAF-treated individuals of the *Prevotella* enterotype exhibited the highest relative abundance of Actinobacteria, with a mean abundance of 3.2% compared to the DTG-treated group (1.7%) and other ART regimens (0.4%) (**Figure 8A**). However, no significant differences were observed in individuals with a *Bacteroides*-dominated gut microbiome in the phylum Actinobacteria (**Figure 8A**). A genus of *Paraprevotellaceae* family was the only genus of individuals with a *Prevotella* enterotype that exhibited significant differences between different treatment groups, and [Eubacterium] was the only genus that exhibited significant differences in the *Bacteroides* enterotype (**Figures 8B, C**). The functional differences linked to ART were minor and insignificant for any possible comparison between the two ART regimens.

## 4 Discussion

In the present study, we analyzed the gut microbiome in PLWH treated with different ART regimens, which has been previously linked to an elevated risk of increased weight gain. We observed the largest differences in gut microbiome composition associated with sexual orientation. The majority of MSM individuals exhibited a *Prevotella*-dominated enterotype, which displayed large differences in composition compared to the gut microbiome of the majority of non-MSM, who exhibited a Bacteroides-dominated gut enterotype. Compared with the pronounced differences in the gut microbiome linked to sexual orientation, the differences in the gut microbiome composition associated with weight gain and ART were minor. The class *Actinobacteria* and genus *Bifidobacterium* were linked to weight gain 12 months after ART switch, and these differences were significantly increased only for individuals with a *Bacteroides* enterotype. In addition, differences in the phyla Firmicutes, Verrucomicrobia, and Synergistetes were observed only for *Bacteroides* enterotype individuals when comparing the 15% with the highest weight gain and the 15% with the highest weight loss 12 months after the ART switch. We found differences in the functional capacities of different pathways between losing and gaining weight 12 months after ART switching, including carbohydrate, glycan and lipid metabolism pathways, in *Bacteroides* enterotype and not in *Prevotella* enterotype individuals.

The predominance of the genus *Prevotella* in the majority of MSM in our study is consistent with other studies (Noguera-Julian et al., 2016; Armstrong et al., 2018; Kehrmann et al., 2019; Huang et al., 2024), which reported a high *Prevotella* abundance in the gut microbiome of MSM. HIV infection is associated with differences in gut microbiome composition, including reduced diversity and alterations of individual taxa (Rocafort et al., 2019; Vujkovic-Cvijin et al., 2020; Rocafort et al., 2024). However, the high *Prevotella* abundance of the majority of MSM is independent of HIV infection, which was initially considered to be associated with a high *Prevotella* abundance. The structure of the gut microbiome composition is clearly distinct between the majority of MSM and most non-MSM, which is not only demonstrated by the difference in the gut microbiome between the dominant genera *Prevotella* and *Bacteroides*, but also by the difference in 47 additional genera between both cohorts. Approximately half of the 59 genera that were identified as significantly different between the *Prevotella-* and *Bacteroides*-dominated gut microbiomes in this cohort of PLWH were also significantly different between the *Prevotella-* and *Bacteroides*-dominated gut microbiomes of PLWH in a previous cohort (Kehrmann et al., 2019), suggesting that the alterations in the gut microbiota structure in PLWH are markedly distinct and not limited to the differential abundance of the major genera *Prevotella* and *Bacteroides* in MSM individuals. Human enterotypes, stratified by the dominant genus and co-occurring other bacteria, were first described by Arumugam et al. (2011) and have been repeatedly described in human gut microbiome studies. In adults, the gut microbiome is generally considered to be stable over long periods in the absence of major disturbances (Faith et al., 2013). Several factors can influence its composition, including socioeconomic status, geographic and ethnic origin, lifestyle, health status, diet, and medications (Rothschild et al., 2018; Scepanovic et al., 2019). Although changes in the gut microbiota composition of individual species can be detected after short periods of dietary changes (Wu et al., 2011), the enterotype remains unchanged and stable in adults in both short- and long-term dietary studies (Wu et al., 2011; Hjorth et al., 2019), including changes to a high-fiber diet for six months (Roager et al., 2014). Individuals from non-Westernized populations and vegan and vegetarian diets in Westernized populations are more likely to be associated with the *Prevotella* enterotype. Nevertheless, predicting the composition of individual gut microbiota remains challenging despite advances in the understanding of the factors influencing the gut microbiota. Several studies have suggested that the *Prevotella*-dominated enterotype observed in many MSM is independent of diet (Noguera-Julian et al., 2016; Armstrong et al., 2018; Huang et al., 2024). In the present cohort, only four individuals were reported to follow a specific diet, of which two individuals were MSM and had a *Prevotella*-dominated gut microbiome and two were non-MSM with a *Bacteroides-*dominated gut microbiome profile. Additionally, four individuals reported having had an antibiotic treatment in the three months before stool sampling, all MSM, of which three had a *Prevotella-* and one had a *Bacteroides*-dominated gut microbiome; therefore, that according to the questionnaire-based answers, a specific diet and antibiotic treatment were not associated with the *Prevotella*-dominated gut microbiome in our study. Sexual practices, including receptive anal intercourse (Vujkovic-Cvijin et al., 2020; Blanco-Miguez et al., 2023) and a high number of sexual partners, have been associated with specific features of the MSM gut microbiome, including a high *Prevotellaceae* abundance and the co-existence of multiple *Prevotellaceae* strains (Huang et al., 2024; Lin et al., 2024).

Many of the differentially observed functional properties associated with the enterotype are related to the metabolism, synthesis, and absorption of sugars, which may support the availability of certain nutrients that influence and promote the structure of certain enterotypes. Only four individuals of our cohort reported following a specific diet (one vegan, one low-fat/low-carb, one low-carb and one LifePlus diet, which includes food supplements and is a nutrient strategy that includes both plant and animal ingredients) so that diet as influencing factor is unlikely responsible for the functional differences observed between the functional gut microbiome characteristics of both groups.

The class *Actinobacteria* and genus *Bifidobacterium* were the only taxa associated with weight gain 12 months after ART switch. These were correlated with weight gain in individuals with *Bacteroides* but not with the *Prevotella* enterotype. Furthermore, a trend towards reduced diversity was observed in individuals with a *Prevotella* enterotype gaining more than 5% weight 12 months after ART change. Reduced Shannon diversity of the gut microbiome is a common finding in individuals with metabolic diseases and has frequently been reported in individuals with obesity and weight gain (Pinart et al., 2021). An increase in the abundance of Actinobacteria in obese individuals has been reported previously (Turnbaugh et al., 2009; Murphy et al., 2010). However, an increased abundance of Actinobacteria, linked to weight gain and obesity, has not been consistently observed. Interestingly, an increase in *Bifidobacterium* abundance has been reported in PLWH with a high BMI undergoing ART (Narayanan et al., 2024). In our study, significance was observed only for the *Bacteroides* enterotype. An increase in the relative abundance of Firmicutes, which was observed in *Bacteroides* enterotype individuals with the highest weight gain and highest weight loss 12 months after ART switch, has been one of the repeated findings of human gut microbiota alterations linked to obesity and has been associated with increased harvest of energy from available food components and with increased nutrient absorption in animal models (Turnbaugh et al., 2006; Jumpertz et al., 2011).

Firmicutes was increased only in the *Bacteroides* enterotype but not in the *Prevotella* enterotype individuals with the highest weight gain 12 months after ART switching in our study. Another feature that was limited to individuals with the highest weight loss after ART switching was the increase in the phylum Verrucomicrobia, which contains *Akkermansia muciniphila* as the most relevant and abundant species. This species has frequently been reported as a next-generation probiotic that contributes to gut health, improves glucose homeostasis, lowers body fat mass, and has been repeatedly associated with weight loss in animals and humans (Everard et al., 2013; Plovier et al., 2017; Yoon et al., 2021).

Interestingly, differences in functional properties between HWG and HWL individuals were observed only in *Bacteroides* enterotypes. The majority of pathways were metabolic pathways, particularly the carbohydrate and lipid pathways, and included pathways such as the phosphotransferase system (ko2060), which was previously shown to be overrepresented in obese individuals (Turnbaugh et al., 2009). Of the 47 KEGG orthologs that differed significantly between the HWG and HWL groups, 45 were associated with the HWG group. Gut microbiomes with a high functional capacity to metabolize complex carbohydrates and lipids may extract higher amounts of energy from the available diet. Overrepresentation of KEGG orthologs involved in lipid metabolism in HWG individuals may facilitate the breakdown and absorption of lipids. However, upregulation of carbohydrate metabolism pathways does not imply a general association with obesity. The degradation of complex carbohydrates by gut bacteria that produce SCFAs, especially butyrate, may also have anti-inflammatory and beneficial functions and health properties, and may be involved in appetite regulation, energy balance, glucose homeostasis, insulin resistance, and protection against obesity (Sanz et al., 2015).

A study investigating more than 1,000 non-antibiotic drugs showed that approximately 24% may have an impact on gut microbiota composition, which differs between individual species (Maier et al., 2018). In addition to the direct effects on microbial growth by inhibiting the growth of some bacterial species in the gut microbiome (Ray et al., 2021), ART may also secondarily affect the gut microbiota composition by suppressing HIV replication, improving immune function, reducing intestinal inflammation, and restoring gut permeability (Villoslada-Blanco et al., 2022). Moreover, the resulting overall improvement in health may also affect appetite and nutrient intake, as well as preferences for food components consumed.

Several studies have analyzed the effects of ART on gut microbiome composition (Villanueva-Millan et al., 2017; Ji et al., 2018; Sortino et al., 2020; Ray et al., 2021; Villoslada-Blanco et al., 2022; Hanttu et al., 2023; Fu et al., 2024; Narayanan et al., 2024). The effects of ART on alpha and beta diversity of the gut microbiome were inhomogeneous between studies that investigated the effects of different ART regimens. However, the restoration of alpha diversity of the gut microbiome by integrase strand transfer inhibitor-based regimens is a repeated finding (Villanueva-Millan et al., 2017; Villoslada-Blanco et al., 2022; Hanttu et al., 2023).

In our study, the differences in gut microbiome composition associated with treatment regimens that increased the likelihood of weight gain were minor. We did not find any significant differences in Shannon diversity associated with ART regimens, including dolutegravir- and bictegravir-containing regimens. The relative abundance of the phylum Actinobacteria was significantly different between ART regimens for the *Prevotella* enterotype samples but not for the *Bacteroides* enterotype samples. Interestingly, we observed few differences in the gut microbiome composition that were associated with the ART regimens, which were observed in *Prevotella* enterotype individuals, in contrast to the observed differences linked to weight gain, which were present in *Bacteroides* enterotype individuals.

The substantial differences in the composition of the gut microbiome between the majority of MSM and non-MSM individuals may confound gut microbiome analyses in cohorts with a high proportion of MSM, which is frequently observed in HIV cohorts. The classification of gut microbiome samples into enterotypes may facilitate the identification of differences linked to variables, such as weight gain or ART, that might otherwise be obscured when analyzing all samples.

Our study was limited by its single-center design. It was not designed to identify biomarkers in the gut microbiome to predict individuals with a higher likelihood of increased weight gain after an ART switch because stool samples were not collected directly before the ART switch. In addition, samples were collected at different time points after the ART switch. The study is also limited by its sample size, which included 118 PLWH. The results of our study should be confirmed by subsequent studies with larger sample sizes, particularly for subgroup analyses of enterotypes associated with weight gain and ART.

## 5 Conclusions

Our study shows that sexual orientation accounts for a significant proportion of the observed variation in the gut microbiome between individuals, with notable differences between the majority of MSM and non-MSM. In comparison, differences in the gut microbiome associated with weight gain and ART treatment in PLWH were relatively minor. However, stratification into enterotypes and separate analyses allowed us to uncover differences in the gut microbiome composition linked to variables such as weight gain or ART that are only present in one enterotype. Analyzing the gut microbiome in the context of the enterotype can improve the identification of microbes that contribute to weight gain or change the composition of the gut microbiome under ART.

## Data Availability

The datasets presented in this study can be found in online repositories. The names of the repository/repositories and accession number(s) can be found below: https://www.ncbi.nlm.nih.gov/, PRJNA1208265.
