# Supplementary figures and images for: Gut microbiota differences linked to weight gain and ART in people living with HIV are enterotype specific and minor compared to the large differences linked to sexual behavior
Source: Front Cell Infect Microbiol. 2025 May 8;15:1568352. doi: 10.3389/fcimb.2025.1568352 (PMC12095285; doi:10.3389/fcimb.2025.1568352)

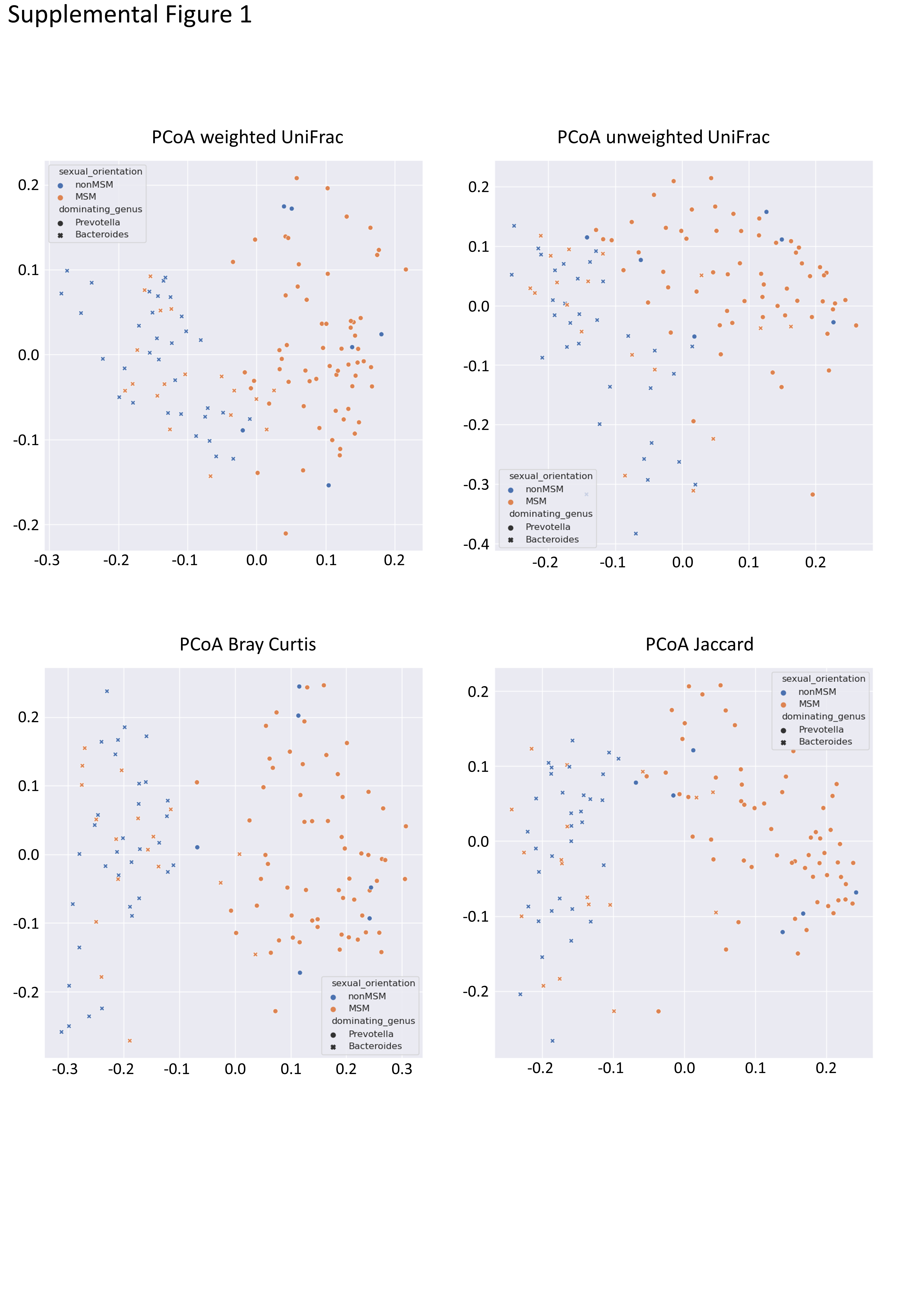

Supplement: Supplementary file 5 [file Image1.tif]
